# The Physicians Visiting List 1873

**Published:** 1872-12

**Authors:** 


*The Physician's Visiting List for* 1873. Philadelphia: Lindsay & Bakiston. For sale by all Booksellers and Druggists.

The Visiting List is almost an indispensable article to a physician, and the one published by Messrs. Lindsay & Blakiston affords every convenience which could be desired by the practitioner. It contains blank leaves for visiting list, addresses of patients, nurses, and others, obstetric engagements, vaccination engagements, &c., and is complete in every particular. They are furnished for from twenty-five to one hundred patients weekly.

